# Advancements in Microprocessor Architecture for Ubiquitous AI—An Overview on History, Evolution, and Upcoming Challenges in AI Implementation

**DOI:** 10.3390/mi12060665

**Published:** 2021-06-06

**Authors:** Fatima Hameed Khan, Muhammad Adeel Pasha, Shahid Masud

**Affiliations:** Department of Electrical Engineering, Lahore University of Management Sciences (LUMS), Lahore, Punjab 54792, Pakistan; 20060029@lums.edu.pk

**Keywords:** artificial intelligence, microprocessors, instruction set architecture, application-specific integrated circuits, real-time processing, machine learning, intelligent systems, automation, multicores

## Abstract

Artificial intelligence (AI) has successfully made its way into contemporary industrial sectors such as automobiles, defense, industrial automation 4.0, healthcare technologies, agriculture, and many other domains because of its ability to act autonomously without continuous human interventions. However, this capability requires processing huge amounts of learning data to extract useful information in real time. The buzz around AI is not new, as this term has been widely known for the past half century. In the 1960s, scientists began to think about machines acting more like humans, which resulted in the development of the first natural language processing computers. It laid the foundation of AI, but there were only a handful of applications until the 1990s due to limitations in processing speed, memory, and computational power available. Since the 1990s, advancements in computer architecture and memory organization have enabled microprocessors to deliver much higher performance. Simultaneously, improvements in the understanding and mathematical representation of AI gave birth to its subset, referred to as machine learning (ML). ML includes different algorithms for independent learning, and the most promising ones are based on brain-inspired techniques classified as artificial neural networks (ANNs). ANNs have subsequently evolved to have deeper and larger structures and are often characterized as deep neural networks (DNN) and convolution neural networks (CNN). In tandem with the emergence of multicore processors, ML techniques started to be embedded in a range of scenarios and applications. Recently, application-specific instruction-set architecture for AI applications has also been supported in different microprocessors. Thus, continuous improvement in microprocessor capabilities has reached a stage where it is now possible to implement complex real-time intelligent applications like computer vision, object identification, speech recognition, data security, spectrum sensing, etc. This paper presents an overview on the evolution of AI and how the increasing capabilities of microprocessors have fueled the adoption of AI in a plethora of application domains. The paper also discusses the upcoming trends in microprocessor architectures and how they will further propel the assimilation of AI in our daily lives.

## 1. Introduction

Artificial intelligence (AI) is a thriving tool that has coupled human intelligence and machine efficiency to excel in various disciplines of life. The idea of building intelligent machines is even older than the field of AI itself. In 1950, Alan Turing presented the possibility of implementing an intelligent machine and gave the parameters to judge its intelligence, known as the Turing test [1]. The term “artificial intelligence” was first mentioned in the Dartmouth Conference in 1956, which was attended by those who later became the leading figures of this field. Early AI research included several programs and methodologies, such as General Problem Solver [2], Theorem Prover [3], natural language processor ELIZA [4] and the discovery of the perceptron [5]. Intrigued by the success of these projects, the Defense Advanced Research Projects Agency (DARPA) also invested massively in this field to utilize machine intelligence for various national security projects [6]. After this first wave, AI encountered technological barriers that curtailed the vast expectations of AI. In the following decades, as the market for personal computers (PCs) was established, the paradigm of AI shifted to a knowledge-based-system, also known as Expert Systems [7]. It was programmed using symbolic programming languages such as LISP or Prolog. Expert Systems were used to implement human expert knowledge in a machine that makes decisions based on stored information [8]. Due to problems in accuracy and efficiency, Expert Systems could not find widespread acceptability in the market.

**Figure 1:**
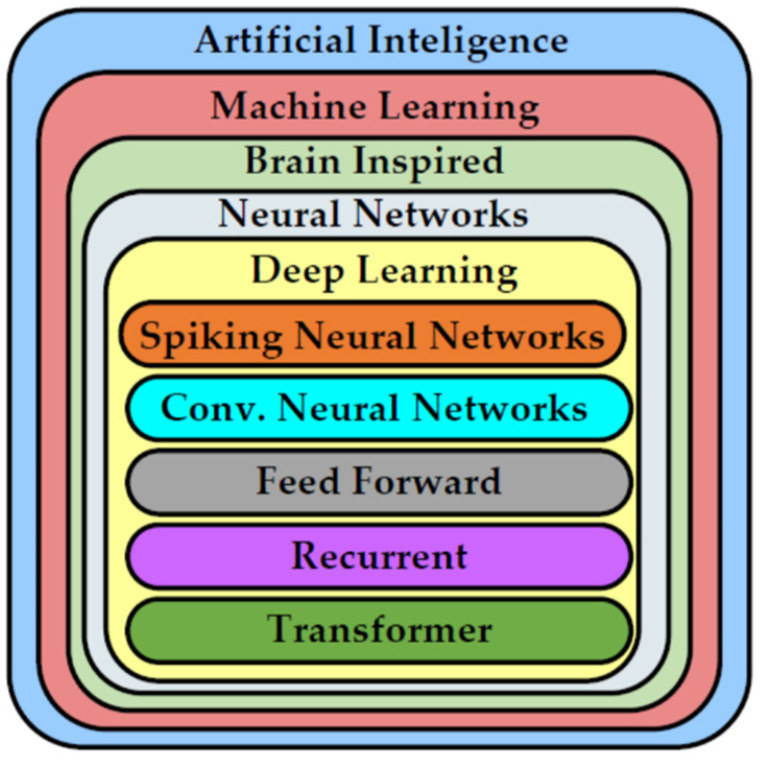
Taxonomy of AI and its sub-fields.

Finally, the increase in computing power and development of more sophisticated mathematical modeling tools gave birth to a new AI paradigm that became more successful than before. The more advanced subset of AI algorithms in the form of machine learning (ML) addressed the complex problems of AI and showed a promising way forward because of its ability to make autonomous decisions based on previous learning and the scenarios at hand [9]. The algorithms used in ML can find applications in diverse use cases, such as the use of decision trees to monitor the depth of anesthesia [10] and support vector machines (SVM) in financial research [11]. Artificial neural networks (ANN) have not only outperformed other ML algorithms but also surpassed human intelligence in specific tasks, e.g., image classification on an ImageNet dataset [12]. It motivated researchers to delve deeper into the ANN structure, which resulted in networks with more parameters, layers, and operations, which came to be classified as deep neural networks (DNN) [13]. DNNs can be further divided into structured network techniques for various applications, i.e., recurrent neural networks (RNN) and transformer networks that mainly focus on natural language processing. In recent years, AI has revolutionized society by covering a wide range of applications, from the simplest smartphones in our hands to security and surveillance [14] and autonomous vehicle controls [15]. Currently, it is being used in agriculture [16], healthcare departments for diagnosing different diseases [17,18], business and finance [19,20], robotics [21], web searches [22], computer vision [23,24], etc. A taxonomy of AI and its various sub-fields is shown in Figure 1.

Initially, limited computing power and memory restricted the growth of AI. Scientists could not go beyond certain performance limits of computers, as the execution time increased exponentially with the algorithmic complexity. Quillian [25] used a vocabulary of only 20 words to present his work on natural language due to computer memory constraints. Even today, when processors are able to deliver 221 K MIPS at 5.0 GHz (Intel core i7-8086 K [26]) compared to the 80 to 130 MIPS in the fastest supercomputer from 1976 (Cray-1 [27]), the architecture for AI applications still demands high resource management to fulfill its real-time requirements. The remarkable research potential of ANN created an appetite for the drastic increase in computational and memory resources as the number of layers in DNNs exceeded 10,000 [28]. Billions of multiply-and-accumulate (MAC) operations necessitated the development of a suitable hardware platform for DNN implementation. It is also worth mentioning that each MAC operation requires frequent memory accesses: three memory reads and one memory write per MAC in worst case, i.e., with no data reuse [9]. Apart from computation and memory requirements, latency is also one of the vital constraints for several real-time applications like self-driving cars, where the slightest processing delay can result in catastrophic consequences [9].

Along with the advancements in intelligent algorithms, the adoption of AI is also driven by the improvement in performance of microprocessors that carry out all the required operations. In 1971, Intel launched the Intel 4004 chip along with its chipset, and the first commercial microprocessor came into being. Intel 4004 [29] was a 4-bit device made up of 2300 transistors and would execute 92600 instructions per second (IPS) at a clock frequency of 740 kHz. Subsequently, a diverse range of microprocessors was developed and the market for computers became ubiquitous. The IBM PC, based on an 8-bit microprocessor (Intel 8088, Intel Corporation, Santa Clara, CA, USA), was introduced in 1979 [30] and became a standard for personal computers (PCs).

Researchers attempted to further increase the processing bandwidth, but the complex micro-architecture became a big hurdle in terms of instruction-set decoding. It led to the idea of reduced instruction set computer (RISC) [31] architecture, which was demonstrated by Acorn RISC Machines (also known as ARM, ARM Ltd., Cambridge, England) based on a 32-bit RISC processor [32]. Driven by Moore’s Law [33], the exponential reduction in transistor size over the years paved the way for 4-bit microprocessors to expand to 64-bit bandwidth while introducing many complex architectural optimizations, i.e., pipelining, cache memory, virtual memory, and application-specific very large instruction words (VLIW) [34]. Major limitations to the implementation of AI were addressed by advancements in different functional modules of microprocessors. For example, an increase in memory bandwidth reduces the operation of frequent data swapping from the memory, which is a major performance bottleneck of AI algorithms, especially for DNNs [35]. AI processors accomplished higher amounts of computing power and reduced memory access time by overcoming the famous power wall [36] and memory wall [37] issues. Still, the general-purpose computers with high-end microprocessors were not able to satisfy the ever-increasing demands of ML and DNN algorithms because of limited number of cores and their monolithic architecture.

Later, graphic processing units (GPUs) equipped with high performance parallelism and memory bandwidth were widely adopted for implementing power-hungry DNN algorithms; this is currently a popular approach for AI implementation [38]. However, in recent years, research in AI architecture has moved towards AI or NN application-specific hardware (commonly known as AI accelerators), which has shown promising results [39]. Many AI-specific accelerators, implemented on FPGA or ASIC, have been proposed to improve energy efficiency, such as DaDianNao [40], PuDianNao [41], Cambricon [42], etc. An innovative industrial approach was developed by Google, called the tensor processing unit (TPU), [43] which reduces the computational precision to accelerate AI operations for the TensorFlow software. Many efficient solutions are emerging for smart embedded processors, but there is room for innovation in this emerging area.

This interaction between the evolution of AI and advancement in microprocessors is following a symbiotic trend that motivated us to write this review paper. Although, many survey papers have been published that give an overview of microprocessor development [44,45] and the emergence of AI [6,9,46] separately, this paper is intended to give an overview of how the progress in microprocessors has enabled the implementation of AI in a broad spectrum of real-life applications. We have restricted this work to the implementation of general-purpose architecture for AI applications because domain-specific hardware is yet an emerging branch of AI [39]. More time needs to pass to evaluate the benefits and directions that AI accelerators will take.

The rest of the of the paper is organized as follows: Section 2 discusses the history and evolution of AI before the exponential improvement in microprocessor capabilities. In Section 3, trends in microprocessors that eventually resulted in the latest accolades in the AI field are elaborated. Section 4 includes a discussion on the confluence of AI and microprocessors and the impact each of these had on the other over the course of the last few decades. Section 5 includes a brief overview of the roadmap followed by trends and the challenges that are expected in future. Section 6 concludes the paper with important observations.

## 2. Origin and Evolution of AI

The idea of making a machine that autonomously performs different tasks has fascinated the human mind even before the advent of digital computers. In 1930, Vannevar Bush presented a set of rules to automatically solve differential equations [47]. After a few years, the British mathematician Alan Turing established the concept of solving any algorithmic problem on a machine that is popularly known as the Turing machine [48]. Practical implementation of AI was made possible by the invention of programmable computers in 1944. Turing’s famous work on measuring machine intelligence gave a jump start to AI. The Turing test [1] is based on the idea of distinguishing between a computer and a human being by observing the conversations between them. In 1952, the first AI program for checkers demonstrated the learning ability of computers [49], and later it was significantly improved by Samuel in 1959 [50] to challenge any decent player of that time. Then, the logic theorist approach was developed using critical concepts of AI to prove some of the geometric theorems [51].

The field of AI officially began with a conference at Dartmouth College in 1956, where the term “artificial intelligence” was first coined by John McCarthy. Many researchers, including the creators of the logic theory machine (Newell and Simon), attended this session and everyone was very curious and positive about the success of AI in the future. The same duo, Newell and Simon, built another AI machine, the General Problem Solver (GPS) [2], and claimed that the GPS could solve any problem given a well-formed description. The GPS, however, did not meet expectations when it came to solving complex problems requiring run-time information handling. It was not that the machine could not solve those problems, but that the time taken to compute answers for complex problems was so long that a machine was impractical. In 1958, McCarthy introduced the first AI programming language, list processing language (LISP) [52] which empowered scientists to store information in the form of objects instead of numbers. Meanwhile, Rosenbaltt’s discovery of the basic unit of neural networks, the perceptron algorithm, gave birth to the concept of connectionism. It was predicted to be the “embryo of an electronic computer,” but further research into this problem was halted due to the work of Minsky and Seymour in 1969 [53] that highlighted severe limitations of the perceptron and Rosenbaltt’s prediction algorithm.

Rapid growth in the research of AI led to the creation of the first ever industrial robot, Unimate, in 1961, to work on assembly lines [54]. Furthermore, the first ever LISP program, known as symbolic automatic integrator (SAINT), to heuristically solve calculus problems was developed by James Slagle [55]. Another approach for mathematical computation called STUDENT was presented by Daniel Bobro [56], which could solve algebra word problems. It is cited as one of the earliest natural language processors, as it was programmed to accept natural language as its input. Later, Joseph Weizenbaum made the first interactive computer program, ELIZA [4], which could hold conversations with human subjects based on some grammatical rules. Weizenbaum was himself surprised to see that many people failed to differentiate between ELIZA and humans. Then, Stanford University produced the first general purpose robot, Shakey [57], which was capable of reasoning on its actions and combined logical reasoning with physical response. The fusion of the two fields, computer vision and natural language processing (NLP), added a new flavor to the field of AI. In a similar vein, another computer program, SHRDLU, was designed to have conversations in English, to plan robot operations, and to apply different actions on simple toy objects [58].

Despite all this success in developing AI algorithms and applications, the most advanced systems were able to handle only a limited scope of problems. In the beginning, the field of AI was perceived with great optimism in all areas of applications. When AI failed to meet expectations, in the 1970s there was a drastic loss of enthusiasm for AI-related research activities both in academia and industry. The main impediment was that AI was unable to overcome the computational barriers in real-time implementation due to the unavailability of powerful processing devices. Scientists had realized that the exponential growth of problem complexity prevented the execution of computer programs in real time. The initial hype of AI attracted many agencies like DARPA, the National Research Council (NRC), and other governmental organizations globally to invest huge funds in this field for different objectives.

Two famous reports, ALPAC (from the US) [59] and Lighthill (from the UK) [60], showed great disappointment in yielding little from AI technology with large investments. Another reason for the drop in AI was the book by Minsky and Seymour, titled “Perceptrons,” in which the authors argued against a learning machine and that only fixed networks were possible. This book had a widespread impact and stopped any further progress in connectionism for almost a decade. Historians called this period an “AI winter”.

**Figure 2:**
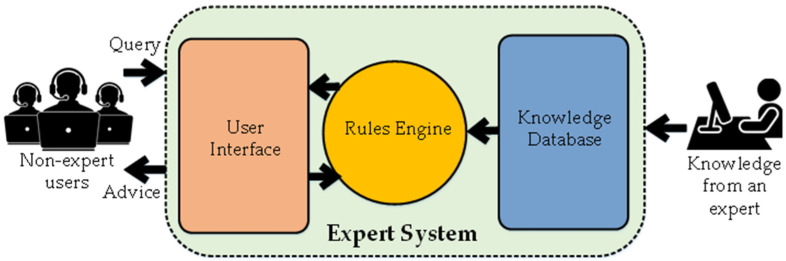
Generic operation of an Expert System.

In the 1980s, the rise of Expert Systems gave new life to AI. The working of Expert Systems was based on a knowledge database with the rules and facts of a particular domain and an inference engine to manipulate the stored symbols [7]. This approach was used to gain human expertise in that specific application. Though Expert Systems became popular in the 1980s, its development had begun in 1965 by Edward Feigenbaum. He built Dendral, which was an Expert System specialized in identifying organic compounds [61]. With passing time, Expert Systems gained commercial attention and MYCIN was built to diagnose blood infectious diseases and prescribe a suitable medicine for them [8]. Another AI programming language, Prolog, was also created in 1972, and mainly focused on linguistic processing [62]. The development of a program, XCON was marked as an enormous success for Expert Systems. It was created for the Digital Equipment Corporation (DEC) to automatically choose computer components according to the requirements. XCON enabled the company to save USD 40 million per year in 1986 [63]. It was the time when Expert Systems were considered to have rejuvenated the field of AI, as they were oriented to solving practical problems at the industrial level. In the late 1980s, many companies were aiming to develop or maintain Expert Systems [64]. Figure 2 shows the generic workflow of an Expert System.

This surge in the field of AI pulled many investments, especially in Japan, where the government dedicated a huge amount of funds to their Fifth Generation Computer System project [65]. Its purpose was to build a machine that could communicate, translate languages, recognize pictures, and argue like a human. This decade also witnessed the revival of connectionism, as John Hopfield proposed a recurrent neural network, Hopfield Net, in 1982 [66]. This neural network offered a remarkable improvement in learning process, which proved to be a hard competitor for the symbolic and logical AI. Rehmalhart et al. [67] also contributed to the revival of neural networks (NNs) by suggesting a backpropagation technique to train NN models. However, it was still too early to implement useful NN applications, as the lack of training data and restricted computing resources ultimately set the limit on its growth.

**Figure 3:**
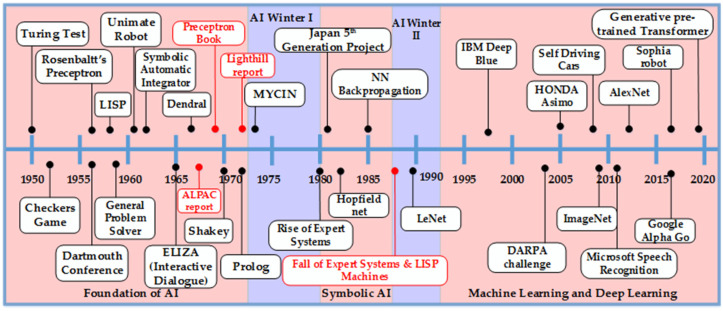
Artificial intelligence over the years.

At the start of the 1990s, Expert Systems also lost steam because of the difficulty in knowledge acquisition and its analysis in real time. The knowledge acquisition process discovered the rules of the real word and linked them to solve any problem in a human manner, but it was not possible to reflect all human skills and feed them into Expert Systems [64]. Moreover, Expert Systems did not have the capability to learn, adapt, and evolve based on their interaction with the users. The situation was worsened by the AI symbolic languages LISP and Prolog, as they led to integration issues in complex systems. The environment of Expert Systems was not compatible with applications programmed in other languages (e.g., C). The rising performance of PCs led to the fading out of Expert Systems, which resulted in a loss of millions of USD for the industries based on Expert Systems [68]. After this downfall, researchers hesitated to put efforts in this field of AI, but many continued to work under different banners such as “machine learning,” “intelligent systems,” and “knowledge-based systems.” The re-branding of all these terms made the survival of AI possible in the future. It also gave fine boundaries to the sub-branches of AI that further evolved with advancements in AI. Figure 3 shows the evolution of AI, going from a simple program solver to deep learning by addressing more and more complex application domains.

## 3. Emergence of Microprocessors

The development of microprocessors is a direct consequence of the invention of semiconductor transistors by Bell Labs in 1947 and the creation of integrated circuit (IC) chips by Robert Noyce in 1961. In 1969, Busicom (Busicom, Osaka and Tokyo, Japan), a calculator company, contacted Intel to order 12 chips for their calculators. In return, Intel came up with four designs, and one of them could be programmed in different ways to fulfill the customers’ requirements. Intel named it Intel 4004 and launched it along with its chipset in 1971. Intel 4004 [29] is known as the first microprocessor, though it was not very powerful, as it could only perform simple 4-bit arithmetic operations. Meanwhile, Texas Instrument (TI) also filed a patent on microprocessors, which was issued in 1973 [69]. TI’s first microprocessor was introduced in 1974, with the name TMS1000, which was also a 4-bit microprocessor with 32 byte RAM and 1 KB ROM. It initiated an avalanche of research and development in this field. Continued interest in improvement in IC technology led to a diverse range of 8- and 16-bit microprocessors within the next few years.

Intel 4004 was followed by the Intel 8008 and 8080 microprocessors. Intel 8080 was an 8-bit microprocessor and the first one to be a part of home-based personal computers. It was later updated to Intel 8085 by adding more instructions, interrupt, and serial input/output (I/O) pins. Motorola also developed its 8-bit microprocessor, the 6800 family, at about the same time. Motorola’s 6800 (Motorola, Chicago, IL, United States) did not play a significant role in minicomputers, but made a great impact on the automotive market [70]. In this way a huge market for embedded processors in the automotive industry was established. The success of Intel 8080 and Motorola’s 6800 led to the development of microcomputers like Atari 2600 (Atari Inc., Sunnyvale, CA, USA), Nintendo Entertainment System (Nintendo, Kyoto, Japan); Commodore 64 (Commodore International, West Chester, PA, USA), Zilog Z80 (Zilog, Milpitas, CA, USA) also came with a DRAM refresh signal and on-chip clock signals to provide better interfacing capability [44]. The increase in acceptance of metal oxide semiconductor (MOS) fabrication technology mainly drove the computer revolution in the 1980s.

The 8-bit architecture of Intel’s 8085 and 8088 was improved to 16 bits in the form of the Intel 8086 microprocessor. The 8086 consisted of bus interface units (BIUs) and execution units (EUs), which were structured to carry out simple pipelined operations wherein the BIU fetched the instructions and EU processed them. The 8086 was also accompanied by a matching floating-point math co-processor chip, 8087, which was based on the implementation of IEEE floating point standard IEEE-754 [71]. Around the same time, the 16-/32-bit Motorola 68000 further advanced microprocessor architecture to fetch the instructions of one or more 16-bit words, which eventually paved the way for a full 32-bit architecture. Another Motorola processor, the 68010, introduced the concept of virtual memory. In 1984, Motorola’s 68020 turned out to be first 32-bit microprocessor with an actual pipeline and an on-chip 256-byte instruction cache.

Thanks to these advancements in processor architecture, computers became accessible to the common person, albeit for use in business and entertainment. Although embedded applications also featured microprocessors, the major requirements of embedded devices in the 1980s and 1990s (calculators, watches, PID controllers, industry displays, etc.) were low cost and convenience of use. Their manufacturers could choose any low-cost microprocessor that gave the necessary performance for that specific application. On the other hand, PC users demanded more variety of applications on the same desktop and got accustomed to utilizing different software libraries. In this scenario, the performance of general-purpose microprocessors became one of the main concerns for customers. The release of continuous updated versions of operating system software and high-level languages, e.g., Windows, C++, Java, etc., also increased the emphasis on the technology of PCs. The Motorola 68000 offered an advanced architecture, but IBM adopted an 8-bit microprocessor, Intel 8088, to power its initial IBM PC in 1981, as it provided a better software interface and utilization with 8-bit peripherals [72]. Not only did IBM establish itself as a leader in the PC market, but also set benchmarks and standards for others.

The development of PCs laid the foundation of the new era for microprocessors. In addition, the evolving complementary metal oxide semiconductor (CMOS) technologies corroborated Moore’s Law and played a vital role in giving shape to new architecture of microprocessors. Mead and Conway’s work [73] on very large-scale integration (VLSI) introduced new design methodologies for academia and industry. Many computer-aided design (CAD) and simulation tools were created, e.g., those from Cadence [74], Synopsys [75], Mentor Graphics [76], etc., to draw schematics and analyze VLSI circuits at various performance levels [73]. It enabled the designers to test new design methodologies and evaluate their performance in the design stage to bring innovations to the architecture of microprocessors. The ever-shrinking geometry of CMOS also helped catalyze the evolution of microprocessors. The transistor channel length deceased to below 1.25 microns in 1985 [77], which resulted in Intel’s 386DX microprocessor, with a gate length equal to 1 micron. It became possible to integrate the entire CPU (excluding memory and floating-point units) on a single chip by the end of the 1980s. The previously used NMOS technology was overruled by CMOS due to its low power dissipation. At that point, the semiconductor vendors were the dominant microprocessor suppliers, who had pretty good knowledge of fabrication and chip making, but lacked intricate details about the internal processor architecture. The future complexities of architecture required specialized knowledge to shift the market to 16-bit architecture as well as embedded microprocessors.

After the mid-1980s, the emphasis shifted to the development of efficient and powerful 16-bit and 32-bit microprocessors as well as corresponding technologies for memory storage and interconnections. Previously, VAX 11/780 by DEC had been a prominent 32-bit mini-computer, but its architecture was based on smaller multi-chip processors. On the other hand, Patterson and Ditzel’s work [31] introduced a new paradigm, called reduced instruction set computer (RISC) architecture, that provided a possible solution to optimize the hardware within given resources as compared to the multi-chip idea of VAX 11/780. The project at University of California, Berkeley, also supported the idea of RISC by designing the Barkley RISC I/II processors [78]. The RISC setup added proper pipelined architecture to microprocessors. For example, RISC I and II used two-stage and three-stage pipelining, respectively. John Cocke’s IBM 801 minicomputer also reinforced RISC concepts on other types of computer organizations [79]. Later, Stanford University took architecture optimization to a new level by introducing a microprocessor without interlocked pipeline stages. They called this millions of instructions per second (MIPS) architecture [80].

Contrary to the previously used complex instruction set computer (CISC) architecture such as Intel 80386, RISC architecture supported 32-bit fixed-length instructions, larger general-purpose registers, and pipelined stages, and avoided memory-to-memory instructions. The simple and regular instruction set for RISC obviated micro-coded ROM, which created more space for full 32-bit instructions. Initially, only smaller OEM accepted the argument about RISC; Acorn Computer in the UK was one of them. They started by amending previously emerging 16-bit microprocessors but were hindered by two major problems: (i) real-time performance in I/O handling and (ii) memory bandwidth utilization. They built their own 32-bit design popularly known as the Acorn RISC Machine (ARM) to overcome these drawbacks [45].

**Figure 4:**
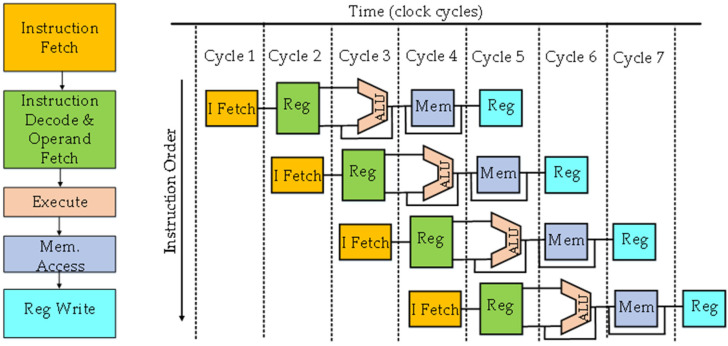
Five stages of pipelining in microprocessors.

Driven by promising claims of RISC architecture, the first commercial RISC CPU, MIPS R2000, entered the market by the mid-1980s [44]. Intel and Motorola also started the development of their own RISC-based microprocessors. A year after the introduction of Motorola 68020, Intel also built its first 32-bit microprocessor, the 80386DX. Motorola 68020 and Intel 80360DX constituted a limited number of pipelined stages. Intel modified its first-generation microprocessors (8086, 80286) by adding new modes of memory addressing, more instructions, and an on-chip memory management unit (MMU). Three years later, Motorola developed 68030, with an integrated MMU and dynamic bus size selection [81]. The increasing number of transistors in microprocessors deepened the pipeline to five stages, as shown in Figure 4, and also incorporated caches and their control functions, the MMU and floating-point unit (FPU), on a single chip. In 1989, Intel used 1.2 M transistors in 80486DX (as compared to 275K in 803868DX) with on-chip FPU [82].

The RISC philosophy and increasing transistor density elucidated the architecture of microprocessors by adding deeper pipelined stages, more on-chip functional units and multilevel caches, higher issue rates, and wider bandwidths [83]. The design of microprocessors was further driven by the race of higher clock rates. It was believed that increasing the clock frequency would directly increase the performance, in line with Moore’s Law. Alpha 21064 was among the first microprocessors to attain a frequency of 150 MHz, and it was followed by Alpha 21164 with 500 MHz [84]. In competition, Motorola, IBM, and Apple collectively designed PowerPC, with a clock speed of 233 MHz. PowerPC adopted a more balanced design scheme as compared to the Alpha series, which compromised the number of instructions and latency for higher clock rates [84]. The desktop market was overtaken by the 32-bit Superscalar Intel Pentium, which sped up many PC-based applications, and its performance limits were further stretched by super-pipelined Pentium Pro. In later years, many versions of the Pentium series were introduced, leading to Pentium III, which pushed the operating frequency beyond 1 GHz [85]. Another company, AMD, giving competition to Intel, developed its first processor, working at 75 to 133 MHz, in 1996 [86]. AMD also jumped in the race of clock rates and released many successors with higher clock speeds. A major advancement was AMD’s Athlon processors, which supported the first 64-bit data path in 2003, and with that the world of microprocessors had reached a new level of performance [86]. At the same time, the embedded market was also developing simultaneously. ARM was quick to cater to this emerging market and established itself as a leader in the mobile handset area through the use of a 16-bit thumb instruction set [87] and easy integration in a system on a chip (SoC) [88]. Table 1 describes some basic features of the popular superscalar architectures of the 1990s and early 2000s.

**Figure 5:**
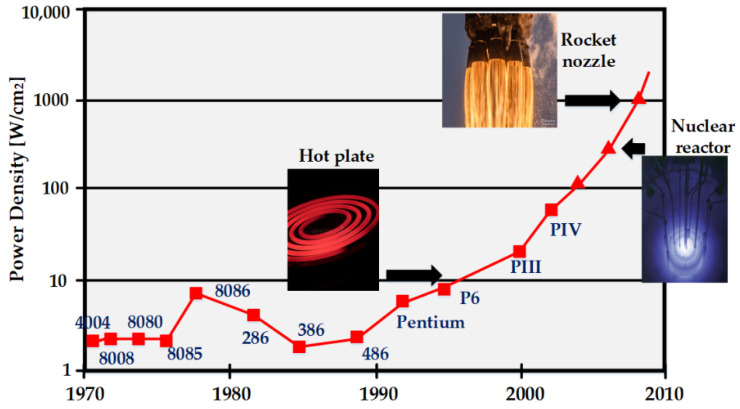
Trend of heat dissipation with the increase in power density of Intel chips.

In the mid-2000s, the interest in the continuous increase in clock frequency was diminished because of power dissipation barriers. Both Intel and AMD strived for smaller feature sizes to achieve high operating frequency, but the chips became too hot and demanded impractical cooling systems for such high transistor densities. Figure 5 represents the predicted trend in heat dissipation levels due to the increase in the power density of Intel chips. Surprisingly, if the chips continued to be manufactured with the same increasing densities, then they could have reached the power dissipation level of a rocket nozzle [89].

Later, the performance bottleneck in microprocessors was overcome by adopting parallel computing techniques using closely coupled multiple processor cores on a single chip [90]. In 2005, AMD released its first dual core processor, Athlon 64 X2 [86]. Intel also rushed into multi-core design and came up with the Core-2 Duo processor for desktop and laptop computers. Some processors, like the Intel Core-2 Duo, were designed with a shared level-2 (L2) cache, whereas Intel Pentium-D and AMD Opteron came with a private L2 cache for each core or processor [91]. Multi-processor chip (MPC) architecture further improved to quad and more cores and initiated a new era of performance gains that yielded profitable results in terms of cost, power dissipation, and a corresponding range of new applications [92].

The Intel Core 2 Quad series enhanced the speed technology of desktop and mobile processors by incorporating four cores with greater sizes of cache memory on a single chip. The family of Core 2 Quad was followed by generations of high-end performance processors, Intel Core i-7. The grouping of Core i7 was usually based on its microarchitecture, with the first generation designed on 45 nm technology, and the recent 10th generation is based on 14 nm processing for up to 8-core chips. AMD also launched a series of processors under the name of “Ryzen” in 2017, which mainly featured the 8-core and 16-thread designs. In the following years, new generations of AMD Ryzen, built on advanced semiconductor technology, came to improve the performance, but significant improvement was witnessed in the third generation of the Ryzen brand [93], which was designed using 7 nm technology. In 2020, the Ryzen 5000 series crossed the Ryzen III gen’s performance by providing a 19% increase in instructions per cycle (IPC) on the same technology. Intel marketed the Xeon family of processors, which mainly targets the workstation, servers, and embedded devices. The AMD Ryzen Threadripper beat the Intel in core count by incorporating a maximum of 64 cores on 7 nm technology. The Intel Xeon processors are designed using same architecture as desktop processors, but support higher number of cores, larger RAM size, advanced cache memory, and many other useful features. Table 2 summarizes the basic features of some of the most prominent multicore processors.

**Figure 6:**
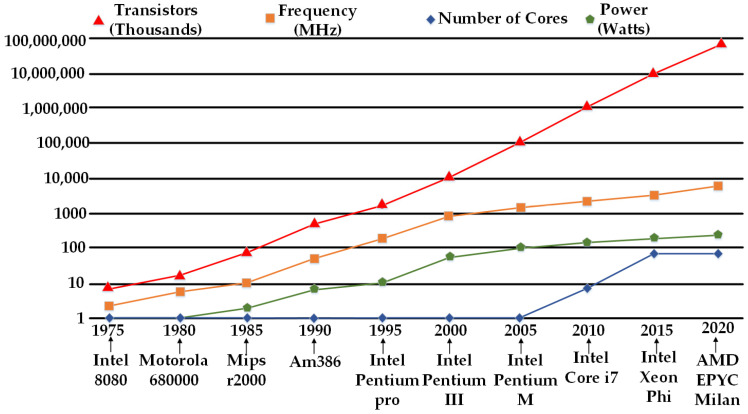
Performance evolution of general-purpose microprocessors.

In tandem, the developments in cache memories, storage technologies, and faster interconnects have provided a support structure that enables microprocessors to provide maximum utilization of advances in their internal architecture to end-user applications [94]. Figure 6 shows the trends in the main characteristics of popular general-purpose CPUs over time. As this paper is aimed at discussing processor architectures, we will not digress, but it is important to mention the significant improvements in associated technologies that allow full performance gains to be obtained from microprocessors.

## 4. Confluence between Artificial Intelligence and Microprocessors

As microprocessors evolved to cater to more complex processing and application domains, the field of AI was also able to overcome many constraints and provide implementable solutions for different challenging tasks. The first practical AI implementation was a handwritten digit recognizer, LeNet [95]. It led to the development of hardware for shallow neural networks, for example, Intel ETANN [96,97], SYNAPSE-1 [98], and ANNA [99]. The AI field flourished under the banner of machine learning (ML), and many self -learning algorithms were proposed to classify images, categorize text, and recognize hand-written characters [100,101,102]. Within the ML domain, neural networks (NNs) became the most popular choice because of their accuracy and straightforward implementation. Deep learning (DL) enhanced the multi-layered architecture of NNs by extracting features at new levels of abstractions [13]. A major breakthrough for DL was seen in the early 2010s, when a large amount of information was incorporated for the training of AI models such as MNIST [103] and CIFAR [104]. The ImageNet dataset was launched about the same time, with more than 3 million labeled images of different categories [105]. Microsoft presented its speech-recognition system based on deep neural networks in 2011 [106]. Further exemplary performances of DL were illustrated by the ImageNet annual challenge [107]. In 2012, the error rate of ImageNet classification dropped to approximately 15% from 25% by utilizing a convolution neural network (CNN)-based model, AlexNet [108]. The classification error rate was further decreased to only a few percent in later years. This success for CNNs in AI implementation shifted the research direction to DL and inspired the development of different hardware platforms for its implementation.

**Figure 7:**
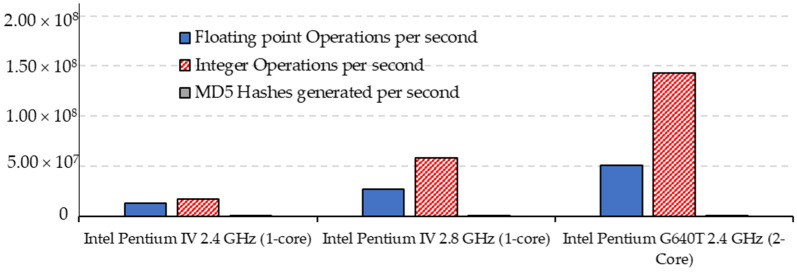
Comparison between CPU operations per second for single core and dual core.

Even though the speed of sequential microprocessors increased drastically over the years, it could only improve performance to a certain extent. The massive computational demands of early AI networks could not be fulfilled by available microprocessors, as some of the AI models needed more than 1 giga floating point operations per second (GFLOPS) to be able to execute in real time [109]. The advancement in the processing throughput of microprocessors came about mainly due to the increase in frequency and most importantly due to the enhanced increase of parallelism. The parallel computation was exploited through multiple instructions issue through pipelining as well as superscalar architectures [90]. Thread level parallelism (TLP) was introduced using multiple processors on a single chip [90]. The boost in performance obtained by multicore processors took the performance gains to the next level, which paved the way for real-time processing of AI applications. Figure 7 shows the comparison between CPU operations per second for single-core Intel Pentium IV 2.4 GHz and Intel Pentium IV 2.8 GHz with dual-core Intel Pentium G640T 2.4 GHz [110]. Intel Core 2 Extreme quad-core provided impressive performance for AI-based applications, especially for gaming on desktops [92]. The multilevel cache memories also contributed to the deployment of AI by providing a faster way to access the memory for huge AI models and large datasets.

**Figure 8:**
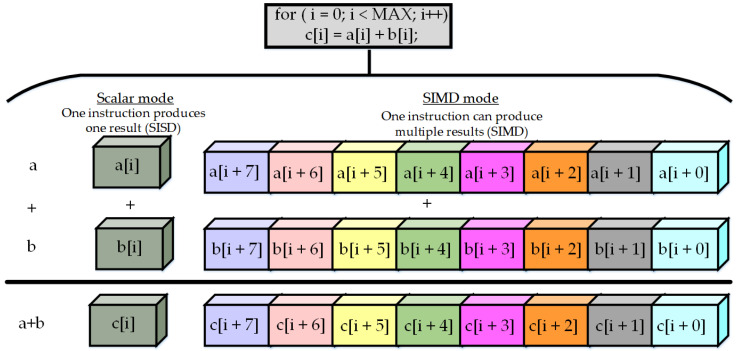
Single-instruction multiple-data (SIMD) operation.

**Figure 9:**
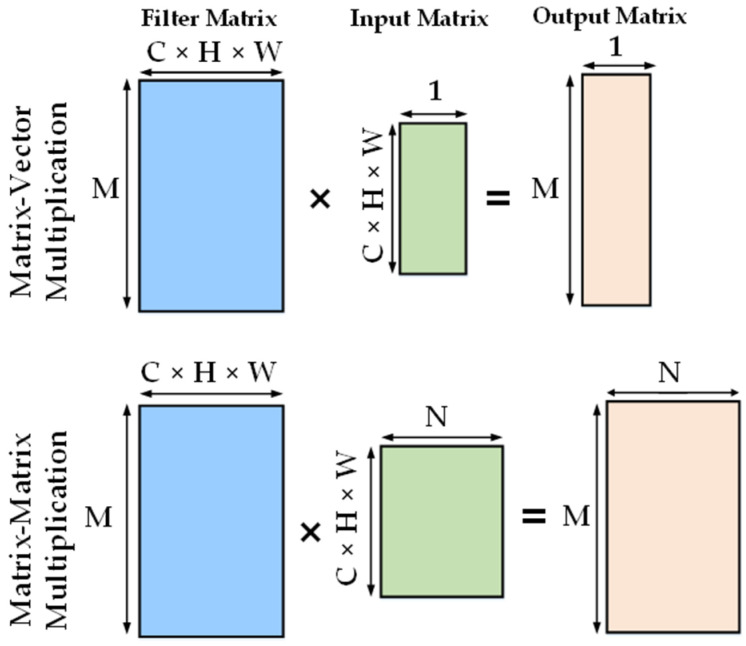
Mapping of fully connected (FC) layers onto matrix multiplication.

**Figure 10:**
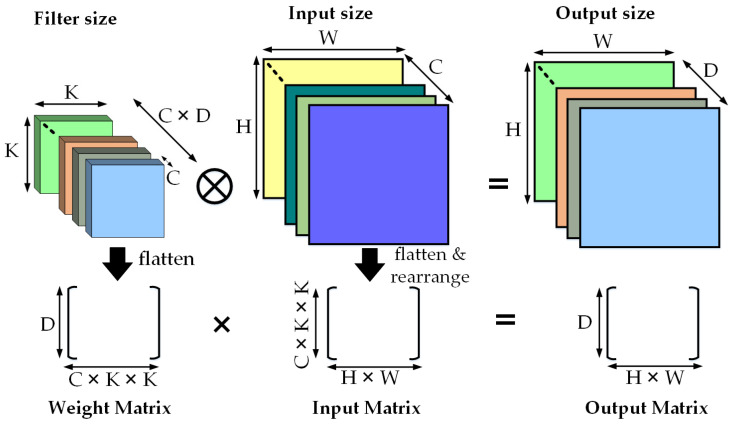
Mapping of convolution (Conv) layers onto matrix multiplication.

The most demanding calculation in CNN computation is the multiply and accumulate (MAC) operation between input and trained weights, which can be performed on multiple data elements simultaneously to achieve faster execution. To facilitate the NN processing, many modern processors feature a special vector instruction set to employ single instruction, multiple data (SIMD). Intel included advanced vector extensions, a 256-bit vector (Intel AVX-256), for each core, which could support the processing of eight single-precision (32-bit) floating point (FP) operations or four double-precision FP operations in a single instruction. Later, Intel AVX-512 increased the size of the vector unit to 512 bits, which doubled the FP operations per instruction [111]. Many updated Intel Xeon processors, like the Xeon Phi series, supported Intel AVX-512 architecture, where they had a designated vector unit per core, as shown in Figure 8 [112]. ARM also launched the ARM Scalable Vector Extension (ASVE) in their modern processors like the ARM Neon [113]. The parallelization technique is further enhanced by mapping fully connected (FC) and convolution (Conv) layers of NNs into matrix multiplications [114,115]. Figure 9 represents the mapping of FC layers as a matrix-vector multiplication with one input feature map with C × H × W = Input_Channels × Height × Width and matrix–matrix multiplication for N number of input feature maps. Figure 10 shows the mapping and arrangement of a Conv layer onto matrix–matrix multiplications. Several compatible optimized software libraries were also launched to support matrix multiplication for DNNs running on these high-performance architectures. Open BLAS (basic linear algebra subroutines) is for multiple microprocessors like ARM, Intel, and MIPS. Intel also developed the Math Kernel Library (MKL) for its processors [116]. There is another popular deep learning framework, called Caffe, for Intel processors [117]. Many tech companies like Facebook and Google have contributed to optimizing other software technologies for Intel architecture for the flexible implementation of modern DNNs.

Intel built Xeon Scalable processors specialized in running complex AI architectures efficiently along with other workloads using the Intel Deep Learning Boost (DL Boost) library. It extended the Intel AVX-512 with Intel inference boost instructions to optimize the vector neural network instructions (VNNI) for deep learning inference. This brought significant performance improvement to image classification, language translation, object detection, speech recognition, and other AI applications in mobile phones, desktops, and servers [118]. Intel DL Boost sped up AI processing by a factor of two, as it used a single int-8 instruction to handle DL convolution, which was previously using three AVX-512 instructions [118]. Previously used 32-bit single-precision FP instructions were converted to 16-bit integer instructions for training and INT8-bit for the inference of DL models with negligible loss in accuracy. Memory access is a bottleneck in the processing of DNNs and also requires a higher order of energy compared to the computational workload [119]. The lower numerical precision not only led to the reduction of memory bandwidth but also helped in efficient utilization of cache memories. Furthermore, it also increased the overall computational throughput [120]. The Intel DL Boost incorporated the brain floating-point format (bfloat16), which enabled a dynamic range of numerical values using a floating radix point [121]. The bfloat16 data type is also included in ARM [122] and AMD [123] microprocessors.

As mentioned above, NN computation is highly parallel in nature, whereas the general-purpose CPUs deal with a wide variety of sequential applications, e.g., binary search trees, data retrieval, string matching, etc. Consequently, graphic processing units (GPUs), which are inherently parallel, became the warehouse for AI processing. Initially, GPUs were used for computer graphics, which is a highly parallel application. The revolution in the architecture of GPUs enabled its use in general-purpose applications. GPUs work efficiently for massively parallel algorithms like AI because of the integration of hundreds (recently, thousands) of cores into one chip [38]. Nvidia realized this potential of GPUs and developed a software library called Compute Unified Device Architecture (CUDA) along with compatible hardware architectures [38]. The Nvidia V100 architecture has 5120 × 32-bit floating-point cores and 2560 × 64-bit floating-point cores, whereas the Intel Xeon Phi family includes 64 to 72 general-purpose cores [124], as mentioned in Table 3 [125]. Due to high computing requirements, AlexNet [108] was implemented with GPUs for the processing of 61 million weights and 724 million MAC operations. It led to the evolution of deeper architecture for convolution networks. A popular DNN called Overfeat adopted the architecture of AlexNet but with a greater number of arithmetic operations (2.8 giga MACs per image) [126]. Another DNN, VGG-16, saw a further increase in the number of weights and MACs of up to 138 million and 15.5 giga operations, respectively [127]. The GoogLeNet comes with a 22-layered architecture with an inception module [128] and it is designed to store all the trained weights in a GPU memory. The multidimensional filters are key to extracting the useful pattern or features of the input data in CNNs, and GoogLeNet uses the filter size of 1 × 1 to reduce the number of weights [129]. There are many updated versions of GoogleLeNet with increased accuracy and corresponding computing cost [130,131].

ResNet went even deeper, with 34 layers [132], and it was the first one to achieve an error rate of less than 5% in the ImageNet challenge. Nvidia’s GPU allowed the implementation of such complex NNs using popular DL frameworks like PyTorch [133], Caffe [117], and Tensorflow [134] through the use of the CuDNN [135] library. The CuDNN library belongs to CUDA-X AI [136], which is a collection of libraries for Nvidia GPUs to accelerate DL and ML. Nvidia’s latest two GPUs (V100 and A100) [137,138] were built with a combination of traditional CUDA and tensor cores. The tensor cores specialize in accelerating the large mixed-precision Matrix MAC operations in a single instruction. The pairing of CUDA and tensor cores enables the Tesla V100 architecture to deliver 120 TFLOPs for DL [137]. The Nvidia A100 GPU enhanced the performance by increasing the number of cores (see Table 3) and supporting numerical formats like INT4, TF32, and others. Tensor format TF32 is a new format to accelerate 32-bit floating-point instructions up to 10 times faster than the V100 32-bit floating-point instruction in DL frameworks. This was further improved 2× by adding a new feature of sparsity in tensor cores [138]. The A100 sparsity pruned the trained weights with the supported sparse pattern and by making an efficient hardware architecture to process the trained weights [138].

**Figure 11:**
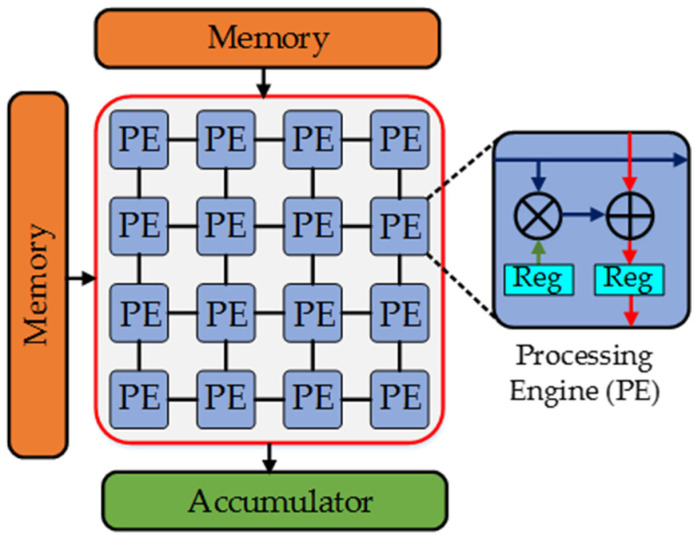
Hardware block diagram showing the generic structure of a spatial architecture.

Recently, technology has been heading towards a dedicated hardware platform for application-specific AI processing. Many DNN accelerators have been proposed and implemented on FPGAs [139] and ASIC [40,41,42] and are usually based on a spatial architecture, as shown in Figure 11. The spatial architecture is designed using dataflow processing through a connected array of processing engines (PEs) (a combination of ALU and its local memory). This two-dimensional (2D) interconnection of PEs facilitates the reuse of the data to reduce the frequency of memory access and increase the parallel computation. The data from the memory can flow left to right and top to bottom through the array of PEs. The PEs are assigned to perform MAC operations on the coming data in a specific manner depending on the dataflow technique.

The data-handling techniques in a spatial architecture can be classified as:

- Weight Stationary (WS): The weights are kept fixed in PEs while inputs flow through the array of PEs with the movement of partial sums. An example includes neuFlow [140] and others [43,141].
- Output Stationary (OS): The accumulation of partial sums is kept constant in PEs to minimize the energy consumption of reading and writing partial sums while broadcasting the inputs and weights to the array of PEs just like in ShiDianNao [142].
- No Local Reuse (NLR): Nothing stays stationary, as the local memory for PEs is eliminated to reduce the area requirement. For instance, DianNao [143] has an NLR dataflow.
- Row Stationary (RS): It aims to minimize the memory access cost by reusing all types of data (weights, inputs, and partial sums) by mapping the rows of convolution on PEs for each sliding window. Eyeriss [144] is one of the accelerators based on RS architecture.

The highlight among all these is an industrial platform called Tensor Processing Unit (TPU), developed by Google. The first TPU was deployed in the Google data center in 2015 [43]. It consisted of systolic arrays of PEs designed for WS dataflow that resembles 2D SIMD architecture. It was followed by another TPU that could process both the training and inference of DNNs in the data center [145]. Later, Google also launched its “edge TPU” for inference in Internet of Things (IoT) applications [146].

## 5. Future Roadmap and Challenges

In the past decade, AI has grown rapidly in its performance and range of applications. It has affected every industry and every human directly or indirectly. Many real-life applications have integrated AI into their functionalities to give exceptional benefits. Still, we are at the beginning of AI in many practical fields. As observed by the current trend in higher accuracy afforded by DNNs, AI algorithms will continue to go deeper into neural structure to attain the capabilities of the human brain to precisely handle critical tasks [147]. Many companies like Apple and Google have huge budgets dedicated to the progress of AI, and academic institutions are also recognizing AI as a distinct field of learning [148]. In this scenario, AI is expected to progress in new and more innovative directions. AI has already given a tremendous boost to several upcoming fields like IoT, big data, autonomous vehicles, and intelligent robotics, and it will continue to drive these technologies in the future. The ongoing revolution of AI will only see an improved uptake in the near future.

Computing capabilities have always been a challenge to the progress of AI. The main focus is on hardware platforms to manage sufficient resources that will be able to fulfill the growing demands of AI. Most of the progress in microprocessors to date has been due to a reduction in transistor size (courtesy of Moore’s Law), but it has already deviated from the predicted performance path, as thermal issues become unavoidable after a certain clock limit. Still, microchips are designed on a very thin layer of silicon wafer, but there some ideas revolving around the three-dimensional structure of microchips to increase the efficiency of microprocessors [149]. Along with benefiting high performance, this concept raises many thermal and interconnectivity issues that researchers need to address before its successful adoption. In other words, microprocessors will prevail with the current trends in architecture for another decade. At the same time, we cannot deny the possibility of novel techniques like quantum computing and molecular computing to change the design of future microchips [150].

Microprocessors have also attained a new level of performance and efficiency due to various parallelization and vector processing techniques that proved to be a driving tool for AI. Considering the limitation of parallel hardware in microprocessors and the deviation from Moore’s Law, it is predicted that the next generation computers will be based on microprocessors working along with highly specialized accelerators dedicated to processing power-hungry AI algorithms. The current research field of AI accelerators will continue to grow and evolve with more advanced trends to make finely tuned accelerators for AI [151]. TPUs are a successful example of application-specific hardware, but it will take some time to adapt the application-specific approach to smart computers, mobile phones, and other embedded applications.

There were huge expectations from AI to change the shape of daily lives, but privacy and security issues are now becoming a major concern in the contemporary era of IoT and big data. There are already hackers out there attacking sensitive industries and data [152,153]. These attacks are expected to increase with the deeper penetration of AI in society through the adoption of smart cities, autonomous vehicles, and intelligent industrial machines. To increase trust in machine intelligence, there is a need for high-performance hardware-enhanced secure technologies. Overall, looking at the evolution of AI since the 1950s, it can be foreseen that the AI field will surmount all these challenges and evolve further in the future with the help from the increasing capabilities of microprocessors.

## 6. Conclusions

The idea of artificial intelligence was initiated way before the birth of microprocessors. For the first 30 years, most AI work was at the algorithmic level. The advent of microprocessors created the need for AI machines, but AI was simultaneously evolving in different directions, and this sluggish progress in the initial years made scientists lose interest in its widespread acceptance. Thus, AI has seen many downfalls during its evolution, but every time it has risen again with new hope and promise. In parallel, the microprocessor was progressing with its dynamics governed by Moore’s Law and MOS technology. The advancements in microprocessor architecture significantly improved its performance and efficiency. The introduction of RISC architecture, superscalar, deep pipelining, and multicore designs gave an exponential boost to the computing power of microprocessors. AI developers soon realized the available computing capability and started to make inroads with machine learning, deep learning and associated datasets. After witnessing the promising results of AI in various applications, microprocessors started supporting AI by amending their architecture in such a manner that they could execute the complex algorithms of AI efficiently for different tasks in the form of SIMD, GPUs, and TPUs. Since then, the two fields, computer architecture and AI, have complimented each other, as microprocessors have adopted different techniques to fuel the growing demands of complex AI models. This confluence between both fields brings AI to every home and industry through PCs, smart gadgets, and other embedded platforms. It can be safely predicted that AI and microprocessor architecture will continue to evolve together in the future with new topologies and dedicated accelerators to deal with challenges like data security and information complexity of data-intensive applications like big data.

## Figures and Tables

**Table 1:** Basic features of popular superscalar microprocessors (1990s–2000s).

High-Performance (Superscalar) Microprocessors					
Microprocessor	Year	Clock Speed (MHz)	Transistor Size (microns)	Cache Size (KB)	Pipe Stages
Intel 486 (Intel, Santa Clara, CA, USA)	1989	25 to 50	0.8–1	8	5
Intel Pentium Pro (Intel, Santa Clara, CA, USA)	1995	200	0.35–0.6	8/8	12–14
DEC Alpha 21164 (DEC, Maynard, MA, USA)	1996	500	0.5	8/8/96	7
Power PC 604e	1997	233	0.25	32/32	6
AMD K5 (AMD, Santa Clara, CA, USA)	1996	75–133	0.35–0.5	8/16	5
MIPS R10000 (MIPS Technologies, Sunnyvale, CA, USA)	1996	200	0.35	32/32	5
Intel Pentium IV (Intel, Santa Clara, CA, USA )	2000	1400–2000	0.18	256	20

**Table 2:** Basic features of popular multicore microprocessors (2005 onwards).

Multicore Microprocessors					
Microprocessor	Year	Clock Speed (GHz)	Transistor Size (nm)	Caches (MB)	Cores
AMD Athlon 64 X2	2005	2	90–65	0.5	2
Intel Core 2 Duo	2006	2.66	65	4	2
Intel Core 2 Quad Q6600	2007	2.4	65	8	4
Intel Core i7-3770	2012	3.4	22	8	4
AMD Ryzen 7 1700x	2017	3-3.6	14	4/16	8
Intel Core i9 10900	2020	5.20	14	20	10
Intel Xeon Platinum 9282	2019	3.8	14	77	56
AMD Ryzen Threadripper 3990X (AMD, Santa Clara, CA, USA)	2020	4.3	7	32/256	64

**Table 3:** Comparison of the number of CPU and GPU cores.

CPU			GPU		
**Processors**	**Minimum Cores**	**Maximum Cores**	**Processors**	**Tensor Cores**	**CUDA Cores**
**Intel Core i7, 10th Gen**	4	8	**Nvidia RTX 2080**	-	4352
**AMD Ryzen**	4	16			
**Intel Core i9, 10th Gen**	8	28	**Nvidia V100**	640	5120
**Intel Xeon Plat. I Gen**	4	28			
**Intel Xeon Plat. II Gen**	4	56	**Nvidia A100**	432	6912
**AMD Ryzen Threadripper**	24	64			
